# P26 User experience of a clinical decision support system in the form of a mobile app in supporting antimicrobial stewardship

**DOI:** 10.1093/jacamr/dlad143.030

**Published:** 2024-01-03

**Authors:** Abiodun Ogundana, Paul Rutter, Sarah Fouch

**Affiliations:** Pharmacy Department, Basildon & Thurrock University Hospital, Mid and South Essex NHS Foundation Trust, Basildon, UK; School of Pharmacy and Biomedical Sciences, University of Portsmouth, Portsmouth, UK; School of Pharmacy and Biomedical Sciences, University of Portsmouth, Portsmouth, UK

## Abstract

**Background:**

There are advancements in the use of technology in modern healthcare. Healthcare professionals use various digital platforms such as electronic medical records, e- prescriptions, websites and smartphone apps to enable prompt access to clinical information. Healthcare professionals are likely to refer to their smartphones for prompt clinical information or guidance that are easily accessible in contrast to paper guides, handbooks and desktop computers. Some studies have shown that mobile application guidelines within a smart phone app increase adherence to prescribing guidelines by clinicians. Furthermore, benefits of a smart phone app are the ability to update them more quickly than paper-based information and the ability to improve clinical decision-making within the form of an electronic clinical decision support system (CDSS). Despite the increased use of digital information there are limited studies in this area on the use of CDSS within smart phone antimicrobial apps. More evidence is needed to better understand healthcare usage of such apps in the area of antimicrobial stewardship. Microguide V1 (M1) is a smartphone antimicrobial app currently in widespread use in UK hospitals designed to provide access to local prescribing guidelines. However, M1 does not have a support tool to ‘direct’ or map prescribers to a specific treatment plan for their patients based on symptoms or suspected diagnosis. Microguide V2 (M2) is an updated version that includes an additional decision support tool that helps to identify the correct antibiotic to prescribe based on patient’s clinical symptoms.

**Objectives:**

To evaluate clinician experiences with both versions of the app within Mid and South Essex University Hospitals (MSE) Trust. In MSE, the M1 is currently in use but M2 is being adopted from November 2023 onwards as part of strategies to improve antimicrobial stewardship, and to help with meeting Commissioning for Quality and Innovation (CQUIN) targets for IV to oral switching of antibiotics).

**Methods:**

A mixed methods approach was devised utilizing an online survey and interviews pre- and post-introduction of M2. Questions were aligned to the COMB-B behaviour model approach influencing change. Topics covered were awareness, experience, barriers, concerns and prescribing behaviour. All prescribers within the Trust working on adult inpatient wards were eligible for participation.

**Results:**

A pre-implementation survey of M2 was conducted between January and October 2023. A total of 473 people accessed the online survey, with 24 responses, but only a total of 15 participants fully completed the survey. The roles of the participants were 6 pharmacists, 3 nurses and 6 doctors. In terms of awareness of the app, 87% stated yes and 38% agreed that the app increased their awareness of antimicrobial stewardship. Two-thirds (67%) of participants used the app in supporting prescribing decisions, whilst a third had never used it. Nearly 40% of participants agreed that the app encouraged them to challenge inappropriate prescribing and almost half (46%) agreed that that they felt comfortable accessing the app at patient’s bedside. The interview analysis is still ongoing.

**Conclusions:**

The results so far provide evidence that M1 is being accessed by prescribers in making clinical decisions and they find it useful. The obvious and major limitation of this study was the small number of responses, and this is partially explained by winter pressures and industrial action by nurses and doctors. This led to reduced availability of participants.

